# Straw blood cell count, growth, inhibition and comparison to apoptotic bodies

**DOI:** 10.1186/1471-2121-9-26

**Published:** 2008-05-20

**Authors:** Yonnie Wu, David C Henry, Kyle Heim, Jeffrey P Tomkins, Cheng-Yi Kuan

**Affiliations:** 1 Department of Genetics and Biochemistry, Clemson University, Clemson, South Carolina 29634, USA; 2 Department of Biosystems Engineering, Clemson University, Clemson, South Carolina 29634, USA

## Abstract

**Background:**

Mammalian cells transform into individual tubular straw cells naturally in tissues and in response to desiccation related stress *in vitro*. The transformation event is characterized by a dramatic cellular deformation process which includes: condensation of certain cellular materials into a much smaller tubular structure, synthesis of a tubular wall and growth of filamentous extensions. This study continues the characterization of straw cells in blood, as well as the mechanisms of tubular transformation in response to stress; with specific emphasis placed on investigating whether tubular transformation shares the same signaling pathway as apoptosis.

**Results:**

There are approximately 100 billion, unconventional, tubular straw cells in human blood at any given time. The straw blood cell count (SBC) is 45 million/ml, which accounts for 6.9% of the bloods dry weight. Straw cells originating from the lungs, liver and lymphocytes have varying nodules, hairiness and dimensions. Lipid profiling reveals severe disruption of the plasma membrane in CACO cells during transformation. The growth rates for the elongation of filaments and enlargement of rabbit straw cells is 0.6~1.1 (μm/hr) and 3.8 (μm^3^/hr), respectively. Studies using apoptosis inhibitors and a tubular transformation inhibitor in CACO2 cells and in mice suggested apoptosis produced apoptotic bodies are mediated differently than tubular transformation produced straw cells. A single dose of 0.01 mg/kg/day of p38 MAPK inhibitor in wild type mice results in a 30% reduction in the SBC. In 9 domestic animals SBC appears to correlate inversely with an animal's average lifespan (R^2 ^= 0.7).

**Conclusion:**

Straw cells are observed residing in the mammalian blood with large quantities. Production of SBC appears to be constant for a given animal and may involve a stress-inducible protein kinase (P38 MAPK). Tubular transformation is a programmed cell survival process that diverges from apoptosis. SBCs may be an important indicator of intrinsic aging-related stress.

## Background

We have observed mammalian cells, in a variety of tissues, transform into individual tubular straw cells in response to desiccation related stress and also occur naturally in live blood and tissues [1]. The transformation event is characterized by a dramatic cellular deformation process which includes: condensation of certain cellular materials into a much smaller tubular structure, synthesis of a tubular wall, growth of filamentous extensions, and the interconnection of tubes to form a tubular network. This tubular transformation occurs constantly and ubiquitously in every tissue we examined, suggesting that this "distinctive trait" involves multiple conserved pathways.

Certain features of the tubular transformation resemble events in apoptosis (the process of programmed cell death that has been well documented [2-4]). The apoptotic process begins with a change in the refractive index of the cell [5] followed by cytoplasmic shrinkage and nuclear condensation. The cell membrane begins to show blebs or spikes (protrusions of the cell membrane), and eventually these blebs and spikes separate from the dying cell and form "apoptotic bodies". At the biochemical level, DNA degradation by a specific endonuclease during apoptosis results in a DNA ladder composed of mono- and oligonucleosomal-sized fragments with 180 base pairs [6]. So far no mutation in the nematode C. elegans, which has long been used in studying apoptosis, has been found to disrupt apoptosis completely [7,8], suggesting the presence of multiple conserved pathways for the same downstream event.

Caspases constitute a large protein family that is highly conserved among multicellular organisms. They are constitutively expressed in most cell types as inactive zymogens that are proteolytically processed before they gain full activity. Because caspases exist as zymogens, their activity is thought to be regulated primarily post-translationally. Inhibition of apoptosis to treat diseases has shown some success [9-11] using Sphingosine 1-phosphate [12] and caspase inhibitors [13-15] in animal models. Many diseases including cancers, autoimmune disorders and neurodegenerative conditions, including Alzheimer's and Huntington's, are believed to be either a failure of apoptosis to eliminate harmful cells or the inappropriate activation of apoptosis, leading to the loss of essential cells.

The production of straw cells from diseased or stressed tissues could conceivably be associated with loss of organ function. The accumulation of a filamentous network of straw cells could also potentially interfere with aspects of the circulatory system. Therefore we undertook a study to more fully characterize straw cells in blood, as well as the mechanisms of tubular transformation in response to stress. Specifically, we wanted to investigate if tubular transformation shares the same signaling pathway as apoptosis. More importantly, the unknown regulation and the number of molecular players in the tubular transformation signaling pathway provides the potential for subsequent development of therapeutics to modulate a variety of human diseases. In fact, we have tested leading small molecule candidates in a screening program to inhibit this type of transformation in both cell lines and in mouse models to help shed light on possible approaches.

## Results

### Straw cell morphologies and straw blood cell count (SBC) in mice and humans

**Figure 1:**
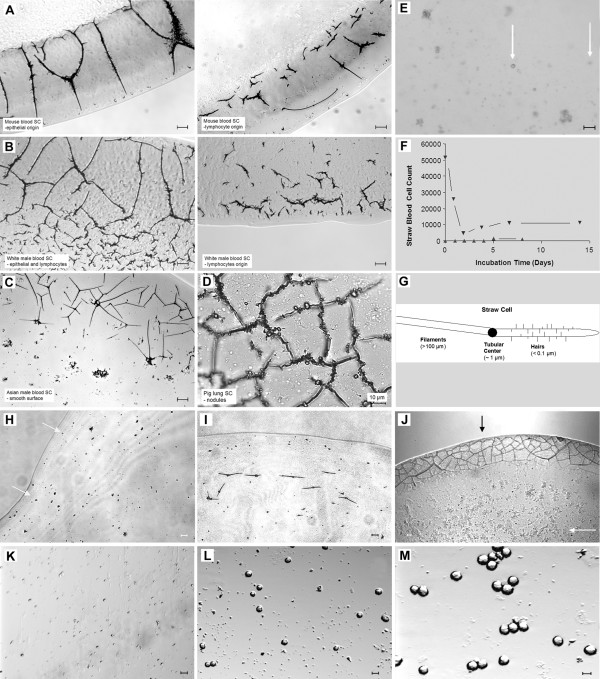
**Straw blood cells in air and in solution.** Straw cells originating from mouse, human and other animals. **A**. Straw cells derived from mouse epithelia, lymphocyte, liver and lung cells. **B **Human filamentous straw cells, **C**. Human smooth straw cells, **D**. Pig lung straw cells, **E**. Straw cells in solution; visible dark dots have diameters near 1 μm (arrows). **F**. Turnover of straw cells incubated at 37°C *in vitro *(▼ Human blood, ▲ Rabbit sera). **G**. Carton presentation of a straw cell and its components. **H**. Synchronized rabbit straw cells in air. Straw cells losing filamentous extensions were incubated in sterile water at day-0. **I**. Emergence of new filaments at day-1. **J**. Emergence of rabbit straw cell network in 5 days (dark arrow), emergence of aggregated cell bodies in solution (white arrow). **K**. Emergence of rabbit regular cells in 6 days, 12 days (**L**) and 14 days (**M**). Space bar equals to 10 μm.

At least five different types of straw cells can be grouped according to their size and morphology. These types include: large straw cells (Figure 1A, first left, from epithelial cells, the curved line is the edge of dried blood droplet), small straw cells with short extensions (from lymphocytes, Fig 1A, middle), straw cells covered with microscopic hairs (straw cells-C and mice, Figure 1B), straw cells with a smooth surface (straw cells-A, Figure 1C) and straw cells stacked with nodules on the surface (Figure 1D, right, from lung cells). In solution straw cells are visible as virtually round shaped dark dots around 1 μm (arrows, Figure 1E). The structure of a straw cell is composed of three major parts: the tubular center with a few filaments and numerous hairs on the surface of filaments (Figure 1G). Straw cells without extensions are shown in Figure 1H.

After analyzing blood drawn from the tail artery of young female mice weighing approximately 30-grams, using 20 mice per experiment in triplicate, we calculated a SBC of (6.2 ± 1.4) × 10^6^/ml. In human adult male blood, we calculated a SBC of (4.5 ± 1.5) × 10^7^/ml (Table 1). The half-life of straw cells (disappearance of filamentous extensions) in blood is 20 hrs at 37°C (Figure 1F). Therefore, at any given time there are approximately 50–100 billion straw cells, that have transformed from regular cells, in human blood (based on a total blood volume of 5–8 liters/person), among which straw cells of lymphocyte origin (with short extensions) account for 40%.

**Table 1:** Straw blood cell properties.

**Animals & Human**	**SBC **(million/ml)	**Blood D. W. **(mg/ml)	**%Straw Cell **(% Dry W.)	Body Weight* (kg)	Lifespan** (Year)
Mouse (*Mus musculus*)	6.2	138	1.6	0.03	1.3
Rats (*Rattus norvegicus*)	16	210	2.7	0.485	3
Rabbit (*O. cuniculus*)	3.5	151	4.3	2.7	5
Chicken (*Gallus gallus*)	3.2	243	2.4	2.9	8
Dog (*D. andersoni*)	1	182	1.0	9	11.5
Sheep (*Ovis aries*)	4.4	169	5.2	50	12
Pig (*Sus scrofa*)	0.6	171	0.6	68	9.5
Human (*Homo sapiens*)	45	229	6.9	75	75
Cow (*Bos taurus*)	1.4	114	2.3	272	10
Horse (*Equus caballus*)	2	177	2.1	544	20

* Body weight was from measurement of individual animals; **lifespan uses average animal lifespan taken from literature.

### Straw cell properties

Straw cells have different properties than the rest of the cells in blood. These cells are extremely hydrophilic and stay intact in a hypotonic buffer. They have filamentous extensions that are often covered with numerous microscopic hairs. Depending on their size and the degree of filamentous extensions, they can be separated from the blood matrix by differential centrifugation and filtration. Straw blood cells from chickens, dogs, rats, and cattle have a large amount of filaments compared to those from sheep, pigs, and horses. Straw cells from humans, mice, and rabbits are the least filamentous. When centrifuged, the hydrophilic interaction with surrounding water keeps straw cells floating at 10,000 rpm. Naked tubular centers (straw cells without filaments) precipitate to the bottom at 5,000 rpm (2, 375 × g). The amount of straw cells in blood by weight was estimated for 9 mammals and one bird (Table 1), with a range from 5.2% for sheep to 0.6% for pigs with an average weight of 3%. The average SBC for the 9 domestic mammals, along with birds and humans was 8 million/ml, with humans and rats leading the counts at approximately 45 and 16 million/ml, respectively.

### Elongation of filaments and enlargement of synchronized straw cells in vitro

Rabbit straw cells isolated from other cells in the sera by differential centrifugation with the addition of methanol (30~50%, depending on the length of filaments for rendering straw cells less hydrophilic) and vortexed vigorously typically lost their fragile filamentous extensions. When viewed under light microscopy these straw cells contained only the tubular center with dimensions just less than 1 μm (Figure 1H). Straw cells isolated from rabbit sera stored at -20°C for over one year were incubated in just sterile water (hypotonic) and they grew new filaments (day 1, Figure 1I). There was a 5-day threshold for the straw cells to grow back into new generations of fully viable straw cells (with filaments) as well as regular cells (arrows, Figure 1J). Based on the number of newly formed straw cells, these were likely the product of old straw cells containing just the tubular center, not the result of regular cells that were left from isolated straw cell populations. The formation of new straw cells from old straw cells leads us to believe that straw cells are not only the product of regular cells but can be formed from other straw cells as well. Treating straw cells with 4% trichloroacetic acid followed by 2 minutes sonication removes their ability to regenerate.

Synchronized straw cells incubated at 22°C and 37°C in 10% medium (DEME, ATCC) showed a filament elongation rate of 0.6 μm/hr for the floating straw cells and an elongation rate of 1.1 μm/hr for those straw cells that were attached to the bottom of the plate (Figure 1I). The enlargement of tubular centers attached to the bottom of the plate, were seen to grow at a rate of 3.8 μm^3^/hr over five days. In two weeks, spherical shaped cells derived from synchronized SBCs without extensions were observed to occupy the bottom of the 4-well chambers (Figure 1K, L, and 1M). This regeneration of spherical cells agrees with our reported observation that CACO2 cells grow back from straw cells to form regular spherical cells during the 30-day incubation period [1].

Minute tubes can be seen aggregating around places where synthesis of new filaments may be happening (Figure 1J). In the zoom-in view, intact straw cells and long stretches of filaments are clearly established in day 8 of rabbit sera incubation. In solution, aggregations of newly emerged spherical shaped cells are seen (Figure 1K and 1L).

### Morphological comparison between apoptotic bodies and straw cells

**Figure 2:**
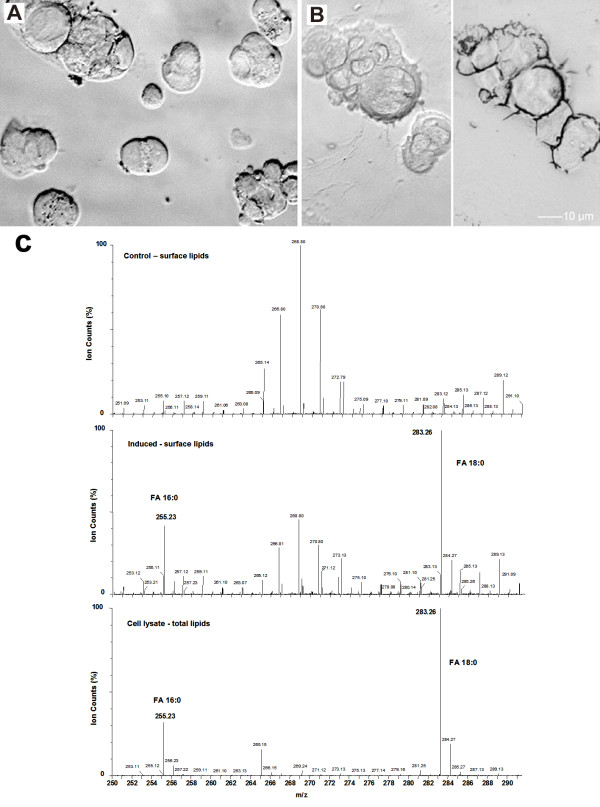
**Production of straw cells *in vitro*.** Straw cells from dehydration induced CACO2 cells *in vitro*. **A**. Normal CACO2 cells, **B**. SC filaments in solution and in air, **C**. Surface and total lipids from normal and induced cells.

*In vitro*, transformed straw cells in solution have a typical apoptosis phenotype in the early stage with the observed membrane blebbing (Figure 2B, left panel). However, in the later stages straw cells begin to differ from apoptotic bodies in the following aspects: (1) they have a continuous tubular structure as opposed to small separate pieces of lower density; (2) growth of filament extensions are invisible in solution but become visible when solution is dry (Figure 2B, right panel); (3) condensation of chromatin in the tubes but not in the remaining cellular debris. The extensions produced during tubular transformation are covered with minute microscopic hairs ([1], Fig 2I). In solution these tubular extensions also appeared to form an interconnected tubular network ([1], Fig 1C). The completion of the tubular transformation is marked by the separation of the discarded plasma matrix from the tube. In apoptosis, the early stages and late stages are marked by an increase in caspase activity, PRPP cleavage, and condensation of chromatin.

The exposure of phosphatidylserine (PS) on the cell surface is a general marker of apoptotic cells [16]. Cell surface lipids in transforming cells were analyzed by mass spectrometry and compared to controls of surface and total lipids in regular cells. Lipid profiling reveals that tubular transformation is a dramatic deformation process. During deformation, the integrity of the plasma membrane is altered so much that free fatty acids, FA 16:0 (255.23 [M-H]^-1^) and FA 18:0 (283.26 [M-H]^-1^) which were absent from the outer leaflet in controls, were detected on the transforming cell's surface. However, changes of phosphatidylserine species, such as PS 36:1 (811.534 m/z), were inconclusive between treatment and controls.

### Signaling pathway comparison between apoptotic body and straw cell

**Figure 3:**
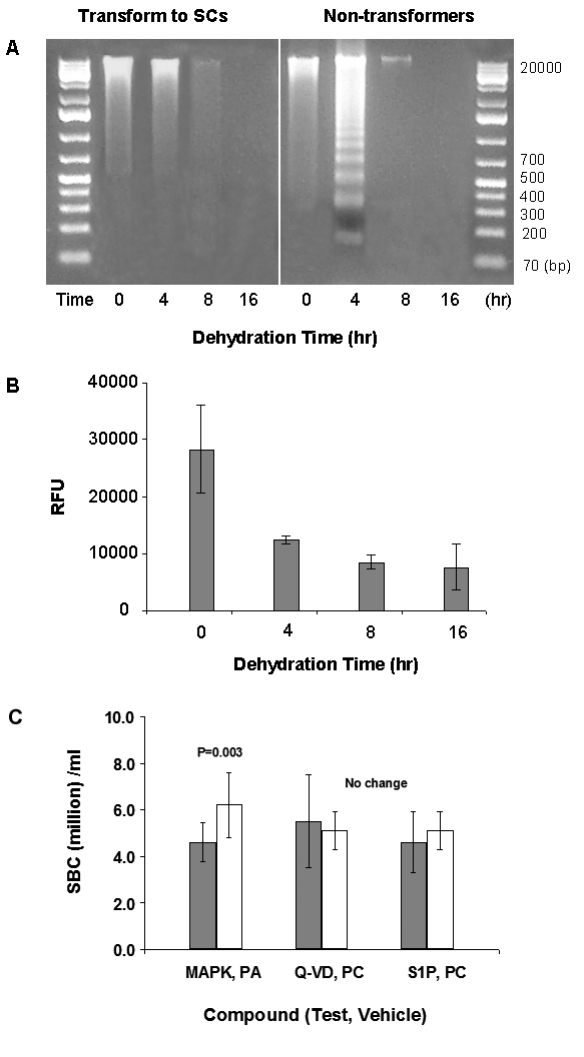
**Comparison of tubular transformation with apoptotic bodies.A**. No appearance of DNA ladder (left panel) in CACO2 cells during a successful tubular transformation induced by dehydration. DNA ladder did appear (right panel) in CACO2 cells during a failed tubular transformation **B**. Measurement of caspase-3/7 activity during dehydration induced tubular transformation in CACO2 cells. **C**. Inhibition of tubular transformation by small molecules in vivo. Blood samples are counted from a single dose sc at 24 hr.

Despite some morphological similarities to apoptosis, the tubular transformation is likely regulated by different pathways in producing the final product. When straw cells are mostly transformed (>90%) no DNA ladder was observed (Figure 3A, left panel). Compared to dehydration conditions which produced no straw cells but instead produced mostly apoptotic bodies (<10% transformation in 1.5× medium), a DNA ladder was observed (Figure 3A, right). Observation of this DNA ladder indicates that in tubular transformation DNA molecules are translocated entirely, opposite to that in apoptotic bodies where DNA molecules are degraded. Caspase 3/7 activity during a successful tubular transformation decreased with the dehydration time (Figure 3B), whereas in apoptosis caspases 3/7 activities increase. In addition, caspase inhibitor (Q-VD-OPh) exerted no inhibition in tubular transformation *in vitro/vivo *either alone or in combinations with other apoptosis inhibitors. At 2 mg/kg body weight, it had no inhibitory activity against tubular transformation and the number of straw cells *in vivo *was not changed compared to controls (Figure 3C and Table 2). A ceramide antagonist, sphingosine 1-phosphate, competing for ceramide mediated apoptosis, had no activity *in vivo *at 1 mg/kg body weight/day and *in vitro *at 200 ppm (Figure 3C, Table 2). Collectively, several lines of evidence suggest that the pathway that leads to tubular transformation in straw cell production diverges from that of apoptosis in apoptotic body production, at least in the late stage. However, the molecular players involved in the signal transduction have not been identified, but the signals are very likely to start at plasma membrane. Study by exerting osmotic stress with PEG (10%), which causes cells to shrink and plasma membranes to separate from the rest of the cell bodies, interrupts the signal and produces no tubular transformation [1].

**Table 2:** Inhibition of tubular transformation by small molecules.

	**CACO2**		**Mouse**		**Note**
**Inhibitors**	μg/ml (ppm)	Tubular T. (Morphology And count)	mg/kg/day (ppm/day)	SBC (%) Reduction	Mode of action (References)
Sphingosine-1-phosphate	200	no inhibition	1	no change	compete for ceramide mediated apoptosis [12]
Q-VD-OPh	200	no inhibition	2	no change	compete for caspase mediated apoptosis [9, 13]
Pyridinyl Imidazole	10	> 90% inhibition	0.01	30	bind to a P38 MAPK Kd = 38 nm [18]

### SBC may play a role in animal aging

**Figure 4:**
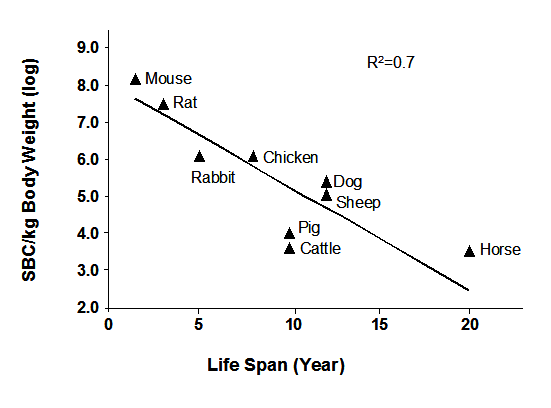
**The relationship between SBC and expected animal lifespan.** The Figure is plotted using data from Table 1.

From nine animals (mouse, rat, rabbit, chicken, dog, sheep, pig, cow, and horse) SBCs are found to correlate with the animal's average lifespan in an inverse relationship with the coefficient of correlation R^2 ^= 0.7 (Table 1, Figure 4). Animals with more straw cells have shorter life spans, suggesting SBC may measure an intrinsic stress level specific to each animal. It was noticed that straw cell production/day/body weight appears independent of age of a given species (data not shown), which agrees with the notion that aging starts at birth and continues at a constant pace [17]. The direct connection between straw cells and the lifespan of an animal suggests that the production of straw cells may cause a loss of cell/organ function in individuals. We speculate this accumulation of straw cells along with the loss of cell/organ function may play a role in many aging related diseases and eventually lead to the death of the individual.

### Inhibition of a stress inducible MAPK reduced straw cells in vitro/vivo

P38 MAPK inhibitor interrupts an environmental stress signal from the cell surface to the nucleus via inhibition of P38 kinase activity. In addition to its promising anti-inflammatory usage [18], we found that it may be involved in the straw cell development pathway. Administration of 0.01 mg/kg body weight/day *in vivo *and 10 ppm *in vitro*, of a pyridinyl imidazole (SB 202190), which inhibits an inducible P38 MAPK, reduced mice SBC by 30% (p = 0.003) in double blind, triplicate experiments with both the test and control using over 20 individual mice. The effects of treatment by three small molecular inhibitors together with controls on SBC are given in Figure 3C (with means and errors) and Table 2. The P38 MAP kinases are a family of serine/threonine protein kinases that play important roles in cellular responses to external stress signals [18]. The resulting reduction in SBC from a treatment with a single dose of SB 202190 suggests that the protein kinase cascade, mediated through IL-1, TNF, IL-18 to MEKK1, 3, NIK, to MKK3, 6 plays a role in tubular transformation.

### Identification of protein involved in the tubulargenesis and regeneration

**Figure 5:**
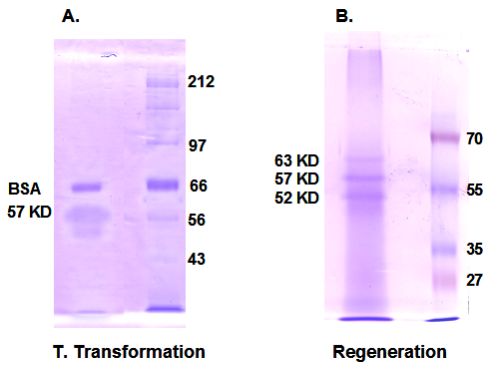
**Identification of proteins involved in tubular transformation and regeneration.A**. Purified tubular proteins on SDS/PAGE in10% acrylamide gel from MCF-7 and CACO2 cells that were transformed into straw cells. **B**. Regeneration of regular cells from straw cells, three proteins at 63, 57, and 52 KD were abundantly expressed in the early stage of the process.

After performing multiple purifications using two types of cancer cell lines, we have purified a unique protein from a culture medium containing an abundance of filaments and bovine albumins (Figure 5). We then performed *de novo *peptide sequencing using a data-dependent acquisition method from digestions of samples with trypsin and chymotrypsin as well as peptide mapping using tryptic peptides and high stringency scores (ladder score and E-value). Using these methods we have tentatively identified a match to a human 57 KD protein known to be a component of the walls of ciliary and flagellar microtubules (Tektin-3, [19]). During regeneration of regular spherical cells from straw cells, three proteins at 52, 57 and 63 KD appear to be abundantly expressed in the early stages of this regeneration (Figure 5, 1B). Micro-sequencing and peptide mapping using tryptic peptides found significant alignment with 80% coverage to a human 57 KD protein (hypothetical, Q8NDG0).

## Discussion

Mammalian blood contains three visible layers when centrifuged. The plasma forms at the top, accounting for ~55% of the volume, then a thin cream-colored layer forms below the plasma consisting of white blood cells and platelets, followed by a final layer consisting of red blood cells, forming the heavy bottom portion of the separated mixture, which accounts for ~45% of the volume [20]. We found straw cells in both the plasma and the bottom layer when centrifuged. The tubular centers are densely packed and heavy. When their filament extensions are lost, they are found in the bottom layer with other lysed cell debris. However, when the filaments are intact, the straw cells remain within the supernatant, even when centrifuged at maximum speed (13,000 rpm or 16,060 × g) on a bench-top centrifuge. At 45 million cells per ml, they account for ~7% of dry mass in human blood.

We compared two methods in straw cell counting: (1) use a Hemacytometer and count the number of dark dots around 1 μm in a defined volume in solution without any distortion to the blood samples and (2) count tubular straw cells from 1 μ of dried blood droplet on cover glass slide directly. We found the numbers derived from two different methods (in solution vs. in air) are in surprisingly good agreement to each other. This agreement suggests that no artifacts were introduced in the counting process and SBCs reflect their actual blood content.

Straw cells occur naturally and at a constant rate in the humans and animals we tested. SBC from nine domestic animals correlates directly to the animal's average expected life span, suggesting a simple connection between tubular transformation and the amount of cells needed to sustain life. In the animals tested, there was an inverse relationship between SBC and the average lifespan of those animals (Figure 4). However, humans appear to have high SBC levels but our average expected lifespan is much higher than the animals we tested and therefore does not fit the trend observed in Figure 4. Perhaps humans, and possibly other primates, exhibit a higher tolerance to elevated SBC levels.

Straw cells may be involved in a variety of human aging-related diseases; for example, invasive malignant tumors contain no distinct boundary between healthy tissue and irregularly shaped cancer cells. Current cancer treatments such as chemotherapy and radiotherapy enhance tumor apoptosis, causing these cancerous cells to transform into apoptotic bodies [21-23]. The strategy of increasing tumor apoptosis is currently being employed in many cancer treatments [24,25]. Our findings predict that this physical and chemical stress not only induces apoptosis but also produces viable straw cells. Straw cells and apoptotic bodies may be the co-products of the same upstream stress responding pathway. We have observed that a DNA ladder, a hallmark of apoptosis, accompanies the tubular transformation (Figure 3A). The hydrophilic nature of straw cells also allows them to flow freely into the blood circulatory system. *In vitro*, straw cells originating from both normal cells and cancer cells germinate and grow back into vegetative cells within a few days to a few weeks. In general, after the remission of the original cancer, the recurrence of tumors in cancer patients frequently occurs within 2~3 years after initial treatments[26,27].

We hypothesize that vast numbers of free circulating cancerous straw cells in blood are capable of regenerating back into actively growing tumor cells at new locations. These circulating straw cells provide a possible mechanism for the establishment of new sites in the body for tumorgenesis. The possibility for straw cells to colonize and grow into tumors may be very small, depending upon the availability of nutrients, DNA mutations, and interactions with the host immune system. However, due to the large number of straw cells present within the blood circulatory system, metastasis could still very well be mediated through straw cells that are produced from increasing physiochemical stresses within a fast growing tumor mass. The straw cells robust cell wall [1] may allow them to tolerate the harsh conditions provided by radiation and chemotherapy. Enabling the straw cells to remain viable even after the original cancer cells, die off (i.e. remission in cancer patients). Cancerous straw cells would then be able to propagate when growth conditions improve *in vivo*.

Since apoptosis was coined in the 1950s, it is believed that programmed cell death is important in keeping cell homeostasis, organ development, and maintaining proper cell population. Although diseases are attributed to abnormal cell death, targeting cancer cells with promoters of apoptosis is an active route of treatment. It is possible that current approaches to cancer treatment involving chemotherapy may contribute to metastasis. Although numerous chemotherapy treatments have been implemented (taxanes, vincas, herceptin, kinase inhibitors, etc.) and they have saved many lives, the delay of a major breakthrough over controlling the disease may be due to the misleading idea that cells die through apoptosis. Where in fact, a portion of the targeted cells enters into a straw cell survival state through "apoptosis" associated stresses to survive and propagate.

We counted the number of straw cells derived from lymphocytes (T and/or B cells) in human blood with characteristic short filamentous extensions to be consistent with the number of lymphocytes that die per day through apoptosis (~50 billion). The SBC in humans is around 45,000/μl, 40% of which are smaller straw cells characteristic of lymphocytes. With a half-life of 20 hr in blood circulation, an estimated 100 billion of all cells are transformed into straw cells every day. Injection of 0.3 μg subcutaneously of a specific protein kinase inhibitor to a 30-gram mouse (equivalent of 1.2 mg to 85 kg person) resulted in a 30% reduction in SBC in the tail artery over 48 hrs. It is unclear if a 30% SBC reduction in mice could be translated to Humans; more studies are needed to make the distinction. The important questions that remain to be answered are: (1) Characterization of the upstream regulatory pathways that promote straw cell formation, 2) Can treatments associated with straw cell reduction significantly improves overall health and longevity? 3) Can straw cell treatment reduction also be an effective way to reduce the spread of tumors in conjunction with other types of treatments?

We have identified one protein involved in the biogenesis of straw cells from the cleavage of purified tubular extensions. This protein can then be targeted for straw cell inhibition, for example, pyridinyl imidazole inhibits the tubular transformation at 10 ppm *in vitro *and 0.01 mg/kg BW/day *in vivo *by inhibiting a stress inducible protein kinase, which may be an early event in the signal transduction.

## Conclusion

Mammalian cells transform into individual tubular straw cells naturally in live blood and tissues in animals and humans, and also in response to increased stresses in CACO2 cells. The transformation event in CACO2 cell is characterized by a dramatic cellular deformation process which includes: nuclei fragmentation and packaging of chromatin into a much smaller tubular structure, synthesis of a tubular wall, growth of filamentous extensions, and the interconnection of tubes to form a tubular network. This tubular transformation occurs constantly and ubiquitously in every tissue we examined, suggesting that this "distinctive trait" involves multiple conserved pathways. Certain features of the tubular transformation resemble events in apoptosis suggesting straw cells may have been studied for decades as products of programmed cell death while in fact they are the products of "programmed cell survival". SBCs may be an important indicator of intrinsic aging-related stress.

## Methods

Pyridinyl imidazole (SB 202190) was purchased from Sigma (St. Louis, MO) Ceramide antagonist, sphingosine 1-phosphate was purchased from Fisher Scientific (Pittsburgh, PA). Caspase inhibitor, Q-VD-OPh was purchased from MP biomedical (Solon, OH). DNA ladder assay kit was purchased from Roche-Applied-Science (Indianapolis, IN).

Blood samples were drawn from young and healthy animals. Female wild type mice, Balb/c, Swiss Webster, between 31–34 g at 120-day-old were purchased from Harlan (Indianapolis, Indiana), Sprague-Dawley rat weighing between 300 – 350 grams at 5.5-month-old were purchased from Harlan (Madison, Wisconsin). SC). Fresh blood samples from Chicken (Gallus gallus, 6 to 8 weeks at 5 to 8 pounds), Sheep (Ovis aries, 2–3 years at 110 lb), Quarter Horse (Equus caballus, 10 years at 1200 lb) were collected from animal farm via Godley-Snell Research Center (Clemson University, SC), Pig (Sus scrofa, 1–2 years at 150 lb) and Cattle (Bos taurus, 1–2 years at 600 lb) from the meat processing lab (Seneca, SC), Dog (D. andersoni, 6 months at 20 lb) from Clemson animal hospital (Clemson, SC), human (Homo sapiens, adults at 160 to 220 lb) from Oconee memorial hospital (Seneca, SC). Rabbit sera were prepared by Cocalica Biologicals, Inc. (Reamstown, PA). Bovine liver was purchased from Bi-Lo grocery store (Clemson, SC). All blood samples were from healthy animals and humans. Cell culturing used DEME medium supplemented with 20% fetal bovine serum (FBS, ATCC) in culture flask in a CO_2 _incubator at 37°C. Chemicals used were from Sigma-Aldrich Company (St. Louis, MO).

### Straw Blood Cell Count (SBC)

Determining SBC used both an in-solution and an in-air method. In solution, a Hemacytometer (SPotlite, Baxter Healthcare Corporation) was used to determine SBC straw cells; visible dark dots with diameters around 1 μm were counted and five random squares were used to average the counts. In the in-air method, 1 μl of dried aliquot placed on a glass slide was used to estimate the SBC count. The numbers from both counts were expressed in SBC/ml. They typically agree with each other within a 10% variation. Fresh mouse and human blood samples, along with ten domestic animals were collected and their subsequent SBC straw cells were immediately determined. Blood samples (264) from 88 mice were counted SBC and averaged. Blood samples from the human frontal arm peripheral veins were used. For other domestic animals, multiple samples (varies from species to species, but at least three samples in each case) were analyzed using 1 μl of blood sample as is, or diluted in 1:10 distilled water to avoid red blood cells in the field. Duplicate readings from each blood sample were averaged and reported.

### Lipid profiling

Lipid profiling by ESI mass spectrometry in negative ion mode uses published protocol [28] Nano-electrospray ionization tandem mass spectrometry (nano-ESI-MS/MS, Q-Tof, Waters) was employed to determine qualitative differences in the lipid molecular species composition between CACO cells undergoing tubular transformation and controls. Lipids were extracted using 200 μl chloroform: methanol = 1:1 to 125000 cells in a four-well chamber that were pre-washed with 1 × PBS to remove any residual lipids from medium. For surface lipids, cells were frozen at (-80°C) and extracted with cold solvents (-20°C) quickly. Four chambers of resulting solvents were combined and concentrated down to 50 μl; NH_4_OH was added to a final 1% solution. For total lipids, cells at room temperature (22°C) were treated with chloroform: methanol: H_2_O = 1:1:1 (200 μl), sonicated and centrifuged. The bottom chloroform phase was collected, combined, and concentrated down to 50 μl. NH_4_OH was added to make a final 1% solution. Acquisition of lipid species used 1 μl sample injected directly to ESI-MS in negative ion mode with a mass scanning range from 200 – 1200 m/z.

### Straw blood cell content

Blood and purified SBC straw cells (mouse, rat, rabbit, chicken, dog, sheep, pig, human, cow, and horse) were estimated for total dry weight by drying out each sample. One ml of sample was dried under a vacuum concentrator (CentriVap concentrator, LABCONCO) until a constant weight was reached. Percentage of straw cell dry weight was estimated by both extraction and filtration methods. First, the sample was centrifuged at 13,000 rpm for 3 min and the supernatant was vortexed twice at 1 min each to break the filaments from the tubular center. The resulting straw cells were centrifuged at 13,000 rpm for 3 min again. Pellets were washed and combined with blood that was extracted twice, using a 1:1:1 solution of chloroform: methanol: water. Next, the top water layer containing straw cells was passed through a 100 KD microconcentrator (Microcon, Amicon) at 5000 rpm for 40 min, twice. Finally, the retained fraction was then loaded on a C18 spin column (Sep-Pak Cartridges, C18 1 cc, Waters) to remove remaining soluble material.

In the filtration method, straw cells were purified using a nitrocellulose membrane. First, whole blood aliquots of 0.5 ml were diluted to 2 ml in distilled water to lyse the regular cells. The sample was then centrifuged to remove cell debris. When blood samples were centrifuged at 10,000 rpm (9530 g) for 3 min, the filamentous tubes moved to the bottom and separated from the naked tubes on top. The tubes without extensions were the densest cellular material, pelleting to the bottom at 5,000 rpm. The aggregated pellet was then washed three times with distilled water. The resulting supernatant, containing straw cells, was combined with previous straw cell fractions and placed in a syringe and pushed through a 0.025 μm nitrocellulose membrane filter (VSWP, Millipore). The amount of soluble material remaining in each passage is governed by (1/4)^n^. Theoretically, the amount of soluble material retained equals 0.02% of starting material when n = 6, which becomes negligible when calculating the dry weight. The n = 8 retained fraction was dried to completion and washed in 80%, cold ethanol (4°C) twice to remove remaining proteins. The resulting dry matter, calibrated by OD 280 nm for protein content was used to estimate the straw cell content. Most rabbit straw cells flow through 0.1 μm membrane (polycarbonate, Solution Consultants^®^).

### Elongation, enlargement and half-life in vitro

Straw cells were synchronized by removing filaments from the tubular center when vortexed in 30% methanol. The straw cells without extensions were collected and placed in 4-well chambers and incubated in 0.1× medium at 37°C. Measurement of newly elongated filaments under light microscope was calculated from ten randomly selected straw cells and measured length in 24 hr intervals over 72 hrs. The results were averaged by total number of hours to give the growth rate in μm/hr. Growth of CACO2 straw cells to regular spherical cells used a synchronized tubular center and measured cell diameters in a 5-day time frame. The results were averaged by the total number of hours to give the growth rate in μm^3^/hr. The experiments were repeated three times. The half-life of straw cells is defined as the loss of the filamentous structure in vitro incubated at 37°C.

### Cell-based assay in 96-well

Compounds mostly insoluble in water were dissolved in medium with help of DMSO, palmitic acid and phosphotidyl choline (PC 36:0) through lipid vesicle hydration methodology [29,30]. Sonication was used to facilitate the solubilization. Lipids in the medium also help solubilizing organic compounds. A known amount of chemical was tested on (1) dehydration induced CACO2 tubular transformation [1] and (2) germination and elongation of CACO2 straw cells. For measuring the inhibitory activity of screening compounds quantitatively, a colorimetric reaction in 96 well format was used that measures the absorbance of total proteins and reducing sugars in THP1 cells that correlates to the amount of intact cells protected from tubular transformation undergoing dehydration treatment. For germination assay, spore-like straw cells were harvested and diluted 10 times in the growth medium and stored at -20°C, 5 ml aliquot of the stock was used for each 96 well plate with 50 μl in each assaying well.

### Preparation of testing molecules in lipid micelle and efficacy study in mice

Injection vehicles were prepared using protocols from the literature [29,31]. Test compounds (a caspase inhibitor, a ceramide antagonist and a pyridinyl imidazole) and placebo (lipid vehicle alone) in various doses were made in 100× stock solution in 1.2 mM palmitate, or PC 36:0 in 1 × PBS solution. Samples were coded randomly. Preconditioned mice (wild type Balb/c, Harlen) were injected subcutaneously with 100 μl of the working solution in 1 × PBS. Blood samples from the tail artery in 5 μl samples were drawn at 0, 24, 48, 72 hr after a single dose. Control (with only injection vehicle) and test samples in doses 0.1, 1 and 10 μmole were injected with 4 mice in each group. The experiment was repeated three times.

Each blood drawing sample containing 5 μl was immediately placed in a collection tube coated with anti-coagulant and diluted in distilled water to 50 μl. Aliquots of 1 μl were placed on a glass slide, dried and counted. The results were analyzed by a Student's T-test (two tailed, equal variance), where means between test and control groups were compared for significance.

### Light microscopy

Cell imaging uses protocol described in the reference [1].

### DNA extraction and Caspase assay

DNA extraction used a protocol described by the manufacturer (Roche Applied Science with modifications. Briefly, 0.5 million cells were harvested and combined in the lysis buffer and combined to have a final volume of 400 μl (200 μl cell plus 200 μl lysis buffer). The final preparation was digested with RNase (RNase A, Ambion) for 30 min at 37°C to remove RNAs bound to the DNA filter. The eluted fraction was concentrated to 30 μl and used 6 μl for DNA electrophoresis. Caspase-3/7 activity was measured according to the manufacturer's menu (Apo-One^® ^homogeneous caspase-3/7 assay, Promega (Madison, WI).

### Protein purification and characterization

Protein purification and characterization used the protocol described in the reference [1]. Synchronized straw cells were incubated in 1× RPMI-1640 medium supplemented with 20% FBS for two weeks at 37°C in a CO_2 _incubator, cells were detached with 0.5% Trypsin at 37°C for 30 minutes. Precipitated cells were dissolved in reducing SDS sample buffer (2 ×) and separated in 10% acrylamide gel at 220 volts. The gel bands were processed by the in-gel tryptic digestion procedure and micro-sequenced by the DDA method (Masslynx, Waters (Milford, MA). Peptide mapping used tryptic peptides by GPMAW software.

## Authors' contributions

YW designed and performed research, wrote the paper, and analyzed data; DCH performed the DNA ladder analysis; KH performed part of the experiment and edited the paper; JPT designed experiment and edited the paper, C–YK prepared cells. All authors read and approved the final manuscript.
